# 547. An Open-label Multicenter Phase 2 Trial of Telacebec for Adults with Buruli ulcer: the TREAT-BU Trial

**DOI:** 10.1093/ofid/ofaf695.020

**Published:** 2026-01-11

**Authors:** Daniel O’Brien, Stephen Muhi, Kathryn Ellis, Bree Sarah, Natasha Savvides, Jenny Henderson, Matt Betteridge, Dean Hickman, Jessica C O’Keeffe, Callum Maggs, Katherine Bond, Kasha P Singh, Maria Globan, Caroline J Lavender, Andrew Buultjens, Timothy Stinear, Majda Benhayoun, Christo H van Niekerk, Eugene Athan, Eugene Sun

**Affiliations:** Barwon Health, Geelong, Victoria, Australia; Melbourne Health, Parkville, Victoria, Australia; Barwon Health - University Hospital Geelong, Geelong, Victoria, Australia; Barwon Health, Geelong, Victoria, Australia; Barwon Health, Geelong, Victoria, Australia; TB Alliance, New York, New York; Tuberculosis Alliance, London, England, United Kingdom; Tuberculosis Alliance, London, England, United Kingdom; Barwon Health, Geelong, Victoria, Australia; Barwon Health, Geelong, Victoria, Australia; Royal Melbourne Hospital, Melbourne, Victoria, Australia; The Royal Melbourne Hospital, Melbourne, Victoria, Australia; Victorian Infectious Diseases Reference Laboratory, Melbourne, Victoria, Australia; Victorian Infectious Diseases Reference Laboratory (VIDRL), Mlebourne, Victoria, Australia; The University of Melbourne, Melbourne, Victoria, Australia; University of Melbourne, Melbourne, Victoria, Australia; Tuberculosis Alliance, London, England, United Kingdom; Qurient Co Ltd, Pretoria, Gauteng, South Africa; Barwon Health, Geelong, Victoria, Australia; TB Alliance, New York, New York

## Abstract

**Background:**

Current WHO recommended treatment for Buruli ulcer (BU), caused by *Mycobacterium ulcerans*, is highly effective but requires a prolonged duration of combination antibiotics that in adults are often poorly tolerated with complex drug interactions, and are associated with prolonged wound healing. Telacebec is a novel, first-in-class antibiotic that demonstrates rapid killing of *M. ulcerans in vitro* and in animal models, and has shown a favorable safety profile in early human studies. It provides the potential for single-drug treatment for BU with reduced duration, side-effects, drug interactions, pill burden and healing times. We aimed to evaluate the safety, tolerability, efficacy and pharmacokinetics of this novel treatment for BU.

**Methods:**

A phase 2 multicenter open-label trial of telacebec 300 mg once daily for 28 days is being conducted at two centres in Victoria, Australia. The study includes adults weighing ≥45 kg with PCR positive *M. ulcerans* lesions that are WHO category I- III if ≤10cm diameter. Primary outcome is complete BU lesion healing rate by 52 weeks after treatment initiation, without relapse and/or curative intent excision surgery.

**Results:**

Forty participants were enrolled from July to December 2024 with a median age of 60 (IQR 44–72) years and 29 (73%) were male. WHO category I, II and III lesions were represented in 32 (80%), 4 (10%) and 4 (10%) participants respectively. Median weight was 85 (IQR 74–92) kg. All participants completed treatment without interruption or discontinuation and there were no drug-related serious adverse events reported. Commonly reported treatment emergent adverse events included headache (18%), lethargy (10%), dizziness (8%), nausea (8%) and diarrhea (8%); all were mild. No patients required surgery. Five (12.5%) swabs taken from lesions after 4 weeks of treatment were culture positive for *M. ulcerans* at 12 weeks incubation; no telacebec associated mutations in the *qcrB* gene were detected and all lesions have healed. All patients have completed at least 34 weeks follow-up. To date 39 (97.5%) lesions have healed with no recurrences.

**Conclusion:**

Early experience suggests that 28 days of telacebec monotherapy may offer a safe, well-tolerated, effective and convenient treatment option for BU.

**Disclosures:**

All Authors: No reported disclosures

